# The database for extracting numerical and visual properties of numerosity processing in the Chinese population

**DOI:** 10.1038/s41597-023-01933-6

**Published:** 2023-01-14

**Authors:** Dazhi Cheng, Zhijun Cui, Chunhui Chen, Xin Xu, Kai Niu, Zhiqiang He, Xinlin Zhou

**Affiliations:** 1 State Key Laboratory of Cognitive Neuroscience and Learning, IDG/McGovern Institute for Brain Research, Beijing Normal University, 100875 Beijing, China; 2 School of Psychology, Capital Normal University, 100073 Beijing, China; 3 Research Association for Brain and Mathematical Learning, Beijing Normal University, 100875 Beijing, China; 4 Department of Pediatric Neurology, Capital Institute of Pediatrics, 100020 Beijing, China; 5 Laboratory of Universal Wireless Communications, Ministry of Education, Beijing University of Posts and Telecommunications, 100876 Beijing, China

**Keywords:** Human behaviour, Perception

## Abstract

The ability to handle non-symbolic numerosity has been recurrently linked to mathematical abilities. The accumulated data provide a rich resource that can reflect the underlying properties (i.e., dot ratio, area, convex hull, perimeters, distance, and hash) of numerosity processing. This article reports a database of numerosity processing in the Chinese population. The database contains five independent datasets with 7459, 4902, 415, 671, 414 participants respectively. For each dataset, all data were collected in the same online computerized test, examination room, professorial tester, and using the same protocols. Computational modeling method could be used to extract the dot ratio and visual properties of numerosity from five types of dot stimuli. This database enables researchers to test the theoretical hypotheses regarding numerosity processing using a large sample population. The database can also indicate the individual difference of non-symbolic numerosity in mathematical abilities.

## Background & Summary

Estimating the nonverbal number of items in a set (i.e., numerosity processing) develops from early childhood^1,2^ and could predict mathematical performance^3,4^. Numerosity processing is usually assessed by a two-dot comparison task, for example, two separate dot arrays^5–8^ or one dot array with two different colors^3^. Both numerical ratio of dot sets (i.e., quantity information)^7,9–12^ and visual features (e.g., area, convex hull, perimeters and distance)^5,13–18^ played an important role in numerosity processing. Firstly, approximate number system (ANS) or number sense theory proposes that the numerical property of dot ratio (i.e., quantity information) is crucial in numerosity performance^7,9–12^. Prior studies have demonstrated that numerosity is represented with magnitude properties that are independent of other dimensions^9–12,19^. The performance in numerosity comparison task becomes better as the ratio between two numerosities increases^20^, which resembles other physical magnitudes (e.g., weight, duration), following Weber’s law^3^.

Secondly, sensory integration system (SIS) theory^15^ or visual form perception theory^21–25^ suggest that numerosity processing is unavoidably influenced by visual properties. Numerosity processing could be modulated by non-numerical perceptual cues such as cumulative surface area^5,14^, contour length^13^, the density of dots^18^, and convex hull^12,26^. For example, modeling studies have demonstrated that the number of dots and their cumulative surface area were perceived holistically, suggesting that the numerical processing of numerosity entails obligatory processing of non-numerical properties^5,13,17^. These findings suggest that visual properties acted as an integral part of the numerosity processing.

Most of the existing studies manipulated the variable of numerosity properties (e.g., dot ratio, area, convex hull, distance) in traditional factor-designed experiments^5,12–14,18,26^. However, these data had relatively small samples and were not publicly available, and how the factor of numerosity properties affects numerosity performance is unclear. Here we report a database, with five large-scale independent datasets (N = 7459, 4902, 415, 671, 414). We also provide the method of extracting the numerical and visual properties (i.e., dot ratio, perimeters, area, convex hull, distance, and hash) from dot stimuli using the computational modeling.

## Methods

### Participants

There were five independent datasets with 7459, 4902, 415, 671 and 414 participants respectively. All participants or their parents read and signed the informed consent before the experiment. These studies were approved by the Institutional Review Board (IRB) of the State Key Laboratory of Cognitive Neuroscience and Learning at Beijing Normal University. They were performed according to the relevant guidelines and regulations.

### Database

**Fig. 1:**
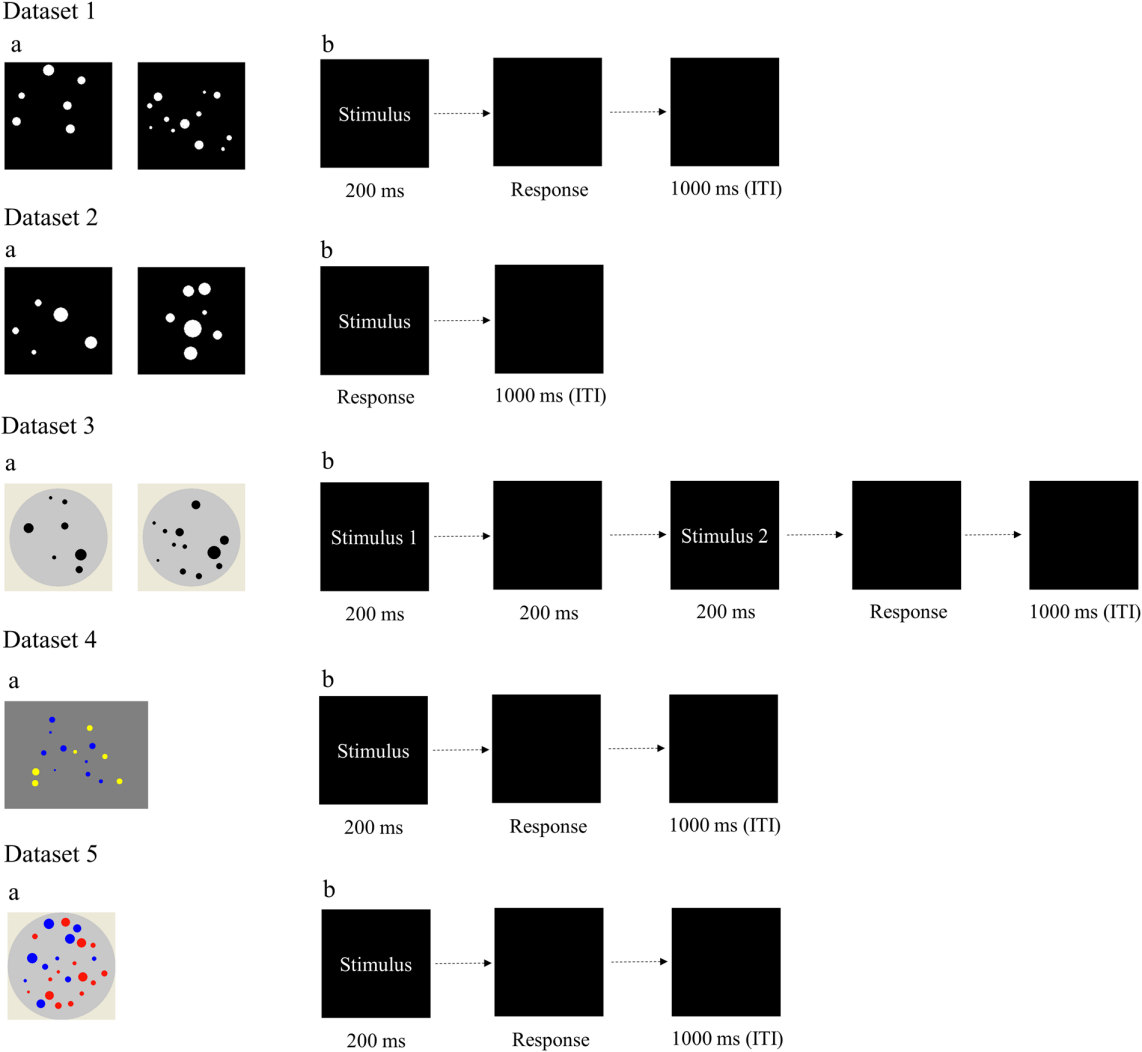
Sample stimuli and task illustration of five numerosity comparison tasks in five datasets. (**a**) the sample stimuli of five numerosity comparison tasks. (**b**) the task illustration of five numerosity comparison tasks. ITI, intertrial interval.

All tasks were programmed using web-based applications in the Online Psychological Experiment System (www.dweipsy.com/lattice). Fig. 1 shows the sample stimuli and task illustration of the five numerosity comparison tasks. Each task has two sessions: practice session and formal testing session. All tasks have shown acceptable half-split reliabilities, ranging from 0.77 to 0.97 according to previous studies^21–25,27–31^. Five numerosity comparison tasks were introduced in each dataset as follows.

#### Dataset 1

This dataset assembled seven published studies with an identical design^21–23,25,27,28^. In the numerosity comparison task, a pair of white-dot arrays were presented against a black background side by side. The number of dots varied from 5 to 32. The dot ratio in each pair ranged from 1.12 to 2.00. There were 120 dot array pairs. Half of the pairs were controlled to have equal total area; the other half had equal mean dot area. Each pair was presented for 200 ms followed by another black screen that lasted until participants responded by pressing the keyboard, followed by a 1-s blank screen before the next trial. Participants were asked to indicate which array had more dots by pressing the key “P” or “Q” on a computer keyboard. A total of 7459 participants aged 5 to 79 years (3955 males and 3504 females, mean age = 18.2 years) completed the task.

#### Dataset 2

This dataset was also composed of several published sets collected through the online platform with an identical design^30,31^. Thirty-six dot array pairs were used. The number of dots varied from five to 12, and the ratios were 2:3, 5:7, and 3:4. The total dot area in each pair was controlled as 2:1, 1:1, and 1:2 for the larger number dot array versus the smaller number dot array, each with 12 pairs. Each pair was presented on the screen until participants responded or 5000 ms elapsed. This presentation was followed by a blank screen for 1000 ms. Participants were instructed to judge the array that contained more dots by pressing the key “P” or “Q” on the keyboard. There were 4902 participants aged 6 to 60 years (2566 males and 2336 females, mean age = 10.1 years).

#### Dataset 3

The task in Dataset 3 was adapted from TEMA2 and published previously^32^. Two sets of black dots distributed in two circles. The number of dots for each array varied from seven to 14. There were 138 dot array pairs. The total dot area in each pair was controlled as 2:3, 1:1, and 3:2 for the larger number dot array vs. the smaller number dot array. Two dot arrays were presented sequentially on a black screen for 200 ms with a 200 ms interval. The interval between the response and the onset of the next trial was 1000 ms. Participants were asked to indicate which array had more dots by pressing the key “P” or “Q” on a computer keyboard. Participants were 415 college students aged 18 to 22 years (178 males and 237 females, mean age = 20.42).

#### Dataset 4

Yellow and blue dots were mixed with no overlap. There were 100 stimuli. Half had equal total area, and the other half had equal mean dot area. The dot number in the arrays varied from five to 16. Dot arrays were presented for 200 ms. The participants were instructed to indicate the dot color presented in greater quantity by pressing the key “P” or “Q” on a computer keyboard. There were 671 participants aged 7 to 38 years (345 males and 326 females, mean age = 16.6 years).

#### Dataset 5

The task in Dataset 5 was adapted from Halberda *et al*.^3^ and published previously as Dataset 3^32^. Red and blue dots were mixed with no overlap. There were 100 stimuli. The ratio of the total dot area of all stimuli was close to 1:1. The ratio of the two types of colored dots varied from 1: 2, 2: 3, 3: 4, 5: 6, to 7: 8. The number of dots for each array varied from 5 to 16. Dot arrays were presented for 200 ms. The participants were instructed to indicate the dot color presented in greater quantity by pressing the key “P” or “Q” on a computer keyboard. Participants were 414 college students aged 18 to 22 years (177 males and 237 females, mean age = 20.42).

### Pre-processing of behavior data

Behavior performance across participants for each dot array pair (Datasets 1~3) or mixed color dots stimulus (Datasets 4 and 5). The mean error rate was defined as the index of accuracy. Reaction time (RT) was the mean response time of correctly responded trials. The participants whose RTs were plus or minus three standard deviations from the mean, were designated as outliers and excluded from further analysis.

### Extracting of properties

Computational modeling method was used to extract the dot ratio and visual properties of numerosity from five types of relatively independent dot stimuli. All computational modeling analyses were conducted with MATLAB (R2018b, The MathWorks, Massachusetts, US) for Windows. Six types of properties in dot stimulus (i.e., dot ratio, perimeters, area, convex hull, distance, and hash) were extracted. The mixed color dots were arranged in two separate arrays. Thus, the analysis was the same across the five datasets. For the dot ratio of numerosity, we counted the number of dots in the two arrays and calculated the dot ratio as a smaller number divided by the larger one. For the visual properties of dot stimuli, the following indices were calculated. Those from the array with fewer dots were divided by corresponding indices from the array with more dots.

**Fig. 2:**
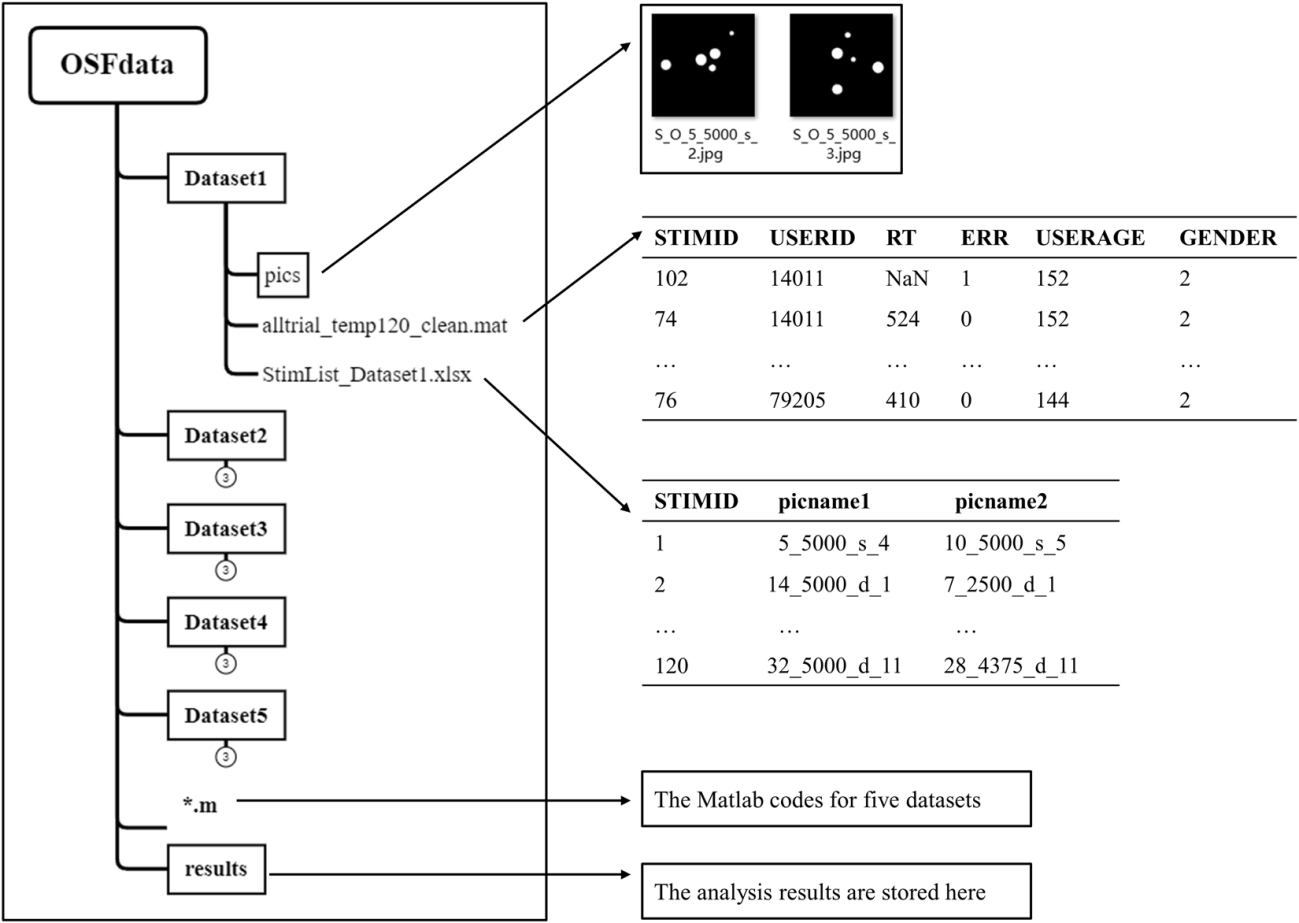
Illustration of the visual properties of dot stimuli.

Five indices of visual properties in dot arrays included: (1) total/mean/standard deviation of areas; (2) total/mean/standard deviation of perimeters (note that some researchers used diameter, which is identical to perimeter, since the latter’s ratio between two arrays is identical to that of diameter, 2πr_1_/2πr_2_ = r_1_/r_2_); (3) convex hull, the area of smallest contour containing all dots; (4) density, the convex hull divided by several dots (Convex Hull/Dot) or total area of dots (Convex Hull/Area); (5) total/mean/standard deviation of the distance between pairwise dots within each array. Fig. 2 showed the illustration of visual properties of dot stimuli.

Each picture was first scaled to 8*8 pixels for visual properties at pixel-level. Then the following hash vectors were calculated. Hamming distance between the two hash vectors of two pictures was calculated. Fig. 2 shows that three-pixel level visual properties in dot arrays included: (1) average hash, calculated by computing bits by comparing whether each color value is above or below the mean; (2) perceptual hash, calculated using discrete cosine transformation (DCT) to get the low-frequency information of the image, then computing the bits by comparing if each DCT value is above or below the median; (3) wavelet hash, calculated by using discrete wavelet transformation (DWT) to get the image’s high-frequency information and then computing the bits by comparing if each DWT value is above or below the median.

## Data Records

**Fig. 3:**
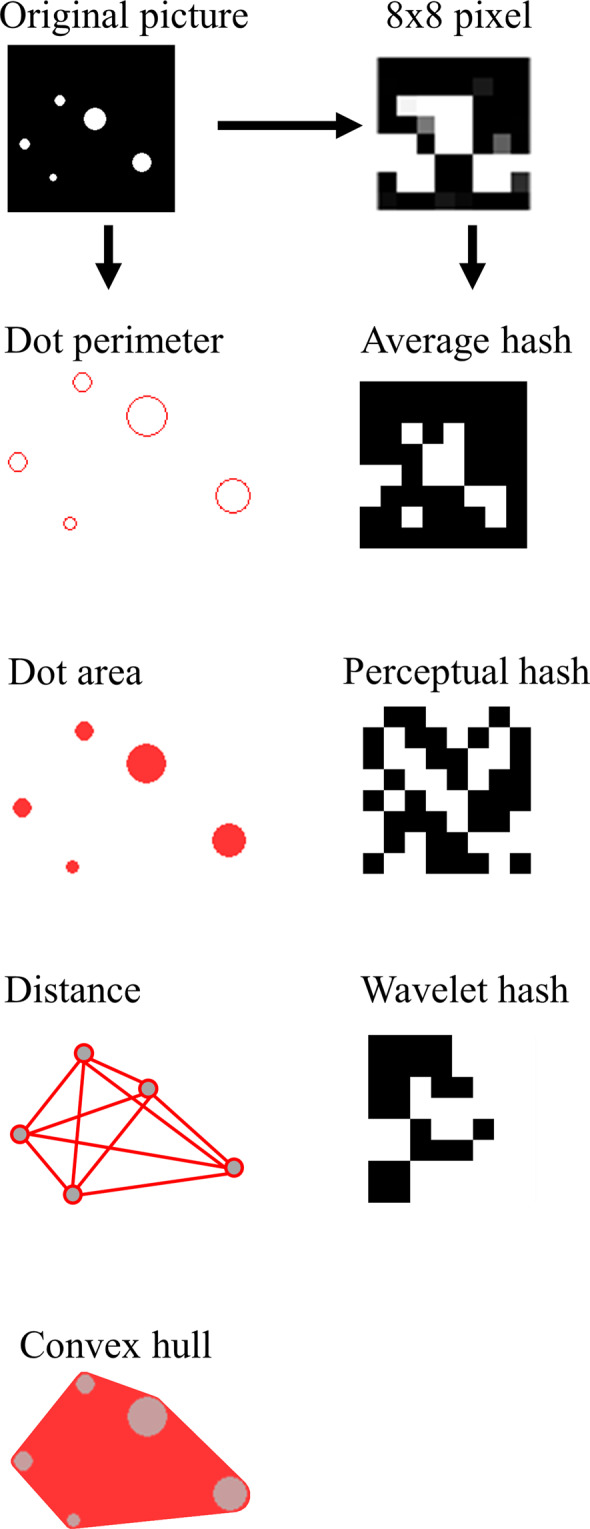
File structure of the repository. The left side shows the overview directory tree of our repository, and the arrows point to the content preview of corresponding files.

The materials and behavioral data of five datasets as well as MATLAB code for analysis are available within the Open Science Framework project^33^ (See Fig. 3).

### Structure of the raw data

The raw data of each dataset were stored separately in Dataset 1~Dataset 5 folder. The subfolder “pics” contains experiment materials used for numerosity comparison task. The “StimList_Dataset*.xlsx” contains the correspondence between STIMID and materials in subfold “pics”. The “alltrial_Dataset*.mat” contained behavioral performance of each participant for each stimuli for Datasets 1–5; detailed information is available as follows:

1. The column named “STIMID” shows the id for each trial;
2. The column named “USERID” shows the id for each subject;
3. The column named “RT” shows response time;
4. The column named “ERR” shows accuracy for response (1: error response, 0: correct response);
5. The column named “USERAGE” shows the age of subject in month;
6. The column named “GENDER” shows the gender of subject (1: male, 2: female).

### Extraction of the numerosity properties

Computational modeling method used to extract the dot ratio and visual properties of numerosity from five independent datasets is shown as follows:

1. “AllStep.m”: the start point of analysis, calls the following code for extracting the numerosity properties;
2. “PicProperty_DatasetAll.m”: Construct the visual properties of 5 dot array datasets;
3. “Subfile_GetViusalProperty.m”: Calculate and assemble properties including dot ratio, area, convex hull, perimeters and distance;
4. “cch_Ahash.m”,”cch_Phash.m”,” cch_Whash.m”: Calculate hash similarity.

### Statistical analyses

“AllStep.m” also calls the following code for all the analysis and result output. Then, steps for statistical analyses and plot results are as follows:

1. Step1_CorrWithDotRatio_DatasetAll.m: Calculate correlations between dot ratio and visual properties;
2. Step2_CombineBehav_DatasetAll.m: Calculate the average error rate (ERR) and reaction time (RT) for each trial;
3. Step3_Regression_DatasetAll.m: Regress each feature on behavior performance to calculate contribution of individual variables (R^2^), as well as the contribution of dot ratio after controlling each visual property (ΔR^2^);
4. FigureA_5dataset.m and FigureB_control1by1.m: Plot regression results.

## Technical Validation

### Qualitative validation

The following criteria assured the data quality of the present database. First, all participants were tested with the computerized test in Online Psychological Experiment (www.dweipsy.com/lattice). Test procedures were presented on a computer screen. Second, all data were collected in an examination room using the same protocols. For each task, standardized instruction was given first, followed by a practice session. After the participant finished the practice session and had no more questions, they could press any key to begin the formal test. Third, each participant was monitored by one tester who was trained to be familiar with the standardized testing procedures. Together, these homogeneities minimize the variation of the experimental environment, tasks, procedures, and participants.

### Quantitative validation

To quantitatively validate the database, we analyzed the contributions of numerical ratio and visual properties to numerosity performance across five independent datasets of dot stimuli. Pearson’s correlation analyses were used to investigate the relationships between visual properties and dot ratio. Hierarchical regression analyses were conducted to examine the contribution of each property, including five visual properties and dot ratio to numerosity performance. Furthermore, the contribution of dot ratio to numerosity performance was also analyzed when indices of visual properties were controlled across five datasets. The ΔR^2^ and corresponding *p*-value are reported.

### Correlation between visual properties and dot ratio

Table 1 shows the correlation of all visual properties with the dot ratio. A Bonferroni correction was used for maintaining the p-value < 0.05 across the 75 correlations. Thus, a conservative p-value of < 0.00067 (=0.05/75) was considered statistically significant. The results showed that the total perimeter’s r value is lower than the total distance for the four datasets.

**Table 1:** Correlation coefficient between visual properties and dot ratio.

Index	Dataset 1	Dataset 2	Dataset 3	Dataset 4	Dataset 5
Total perimeter	0.78^*^	0.13	0.53^*^	0.81^*^	0.93^*^
Mean perimeter	−0.44^*^	−0.02	−0.43^*^	−0.47^*^	−0.95^*^
Std of perimeter	−0.16	−0.35	−0.30^*^	−0.05	−0.15
Total area	0.37^*^	0.01	0.07	0.37^*^	0.42^*^
Mean area	−0.47^*^	−0.08	−0.44^*^	−0.50^*^	−0.98^*^
Std of area	−0.28	−0.36	−0.35^*^	−0.18	−0.31
Convex hull	0.50^*^	0.21	0.46^*^	0.25	0.32
Convex hull/Dot	−0.31^*^	0.03	−0.36^*^	−0.32	−0.26
Convex hull/Area	0.05	0.05	0.26	−0.08	0.3
Total distance	0.95^*^	0.60^*^	0.94^*^	0.88^*^	0.90^*^
Mean distance	0.05	0.1	−0.02	−0.12	0
Std of distance	0.2	0.11	0.06	−0.01	0.15
Average hash	−0.02	−0.04	−0.01	−0.05	−0.11
Perceptual hash	−0.09	0.2	0.13	−0.12	−0.18
Wavelet hash	−0.29	0.32	−0.15	0.03	−0.02

Note. *p < 0.05, Bonferroni-corrected.

### Explained variance of each property to numerosity performance

**Fig. 4:**
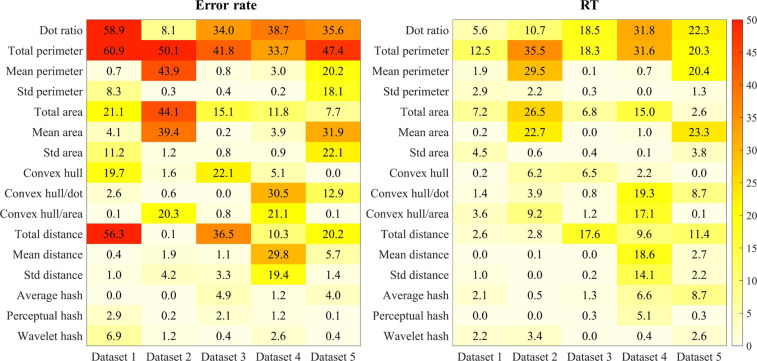
Explained variance (%) of each property to numerosity performance across five datasets. The darker the color, the higher △R^2^ of the property to numerosity performance.

Fig. 4 shows the explained variance (%) of each property related to numerosity performance across five datasets. A Bonferroni correction was used for maintaining the p-value < 0.05 across the 80 regression analyses. Thus, a conservative p-value of < 0.00062 ( = 0.05/80) was considered statistically significant. The dot ratio significantly accounted for the variance of numerosity performance across the error rate of all datasets except for Dataset 2, with all R^2^ > 35.5%, the Bonferroni-corrected *p < *0.05. However, across the error rate of all datasets, the total perimeter accounted significantly for the numerosity performance variance, with all R^2^ > 33.7%, the Bonferroni-corrected *p* < 0.05.

Across the RT of five datasets except for Datasets 1 and 2, the dot ratio significantly accounted for the numerosity performance variance, all R^2^ > 15%, the Bonferroni-corrected *p* < 0.05. Across the RT of all five datasets, the total perimeter accounted significantly for the numerosity performance variance, with all R^2^ > 12%, the Bonferroni-corrected *p* < 0.05.

### Contribution of dot ratio to numerosity performance when controlling for visual properties

**Fig. 5:**
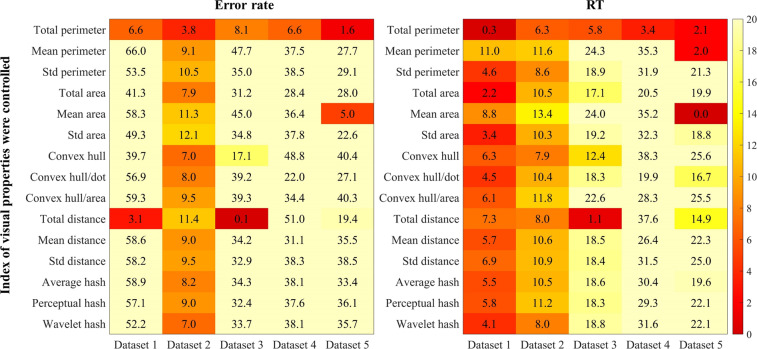
Unique contribution of dot ratio (△R^2^) to numerosity performance when indices of visual properties were controlled across five datasets (each visual property entered regression first, followed by dot ratio). The number of five datasets was used as dummy variable. Std: standard deviation. The darker the color, the lower △R^2^ of dot ratio to numerosity performance after controlling for visual properties.

We performed multiple hierarchical regression analyses (see Fig. 5) to examine the contribution of the dot ratio to numerosity performance, when controlling for visual properties. A Bonferroni correction was used for maintaining the p-value < 0.05 across the 75 regression analyses. Thus, a conservative p-value of < 0.00067 ( = 0.05/75) was considered statistically significant. Across all datasets, when controlling for total perimeter, the dot ratio no longer accounted for the variances of RT in numerosity performance, all ΔR^2^ < 6.3%, the Bonferroni-corrected *p* > 0.05. When controlling for some visual properties, including area, convex hull, and hash, the dot ratio significantly accounted for the variances of error rate or RT in numerosity performance in Datasets 3, 4, and 5.

## Usage Notes

The current database is available on the OSF repository. All codes for preprocessing, computational modeling and plotting are openly accessible. This database can contribute to understanding the contribution of numerical ratio and visual properties to numerosity processing. First, the current database can be analyzed to test the theoretical hypotheses regarding numerosity processing. Second, it can be used to find the optimal properties for new computational models of numerosity processing and can provide benchmark data to evaluate them. Third, the current database, combined with the existing databases of numerosity processing in Western countries, can be used to examine how visual perception affects numerosity cross culture. Finally, the large-scale numerosity measures reported in the database can be calculated to the normative score for numerosity performance. It could be served as the norms of the individual difference in numerosity performance such as non-symbolic numerosity deficits. Thus, it can be useful to scientific research to investigate the individual difference in non-symbolic numerosity processing.

## Data Availability

The codes used to preprocessing the data, calculation of numerosity properties and plot results are openly available on the OSF repository^33^. For more details about code usage, please refer to the OSF repository.
